# Shall We Give up the Novel?

**Published:** 1887-05-07

**Authors:** 


### Shall we give up the Novel?

By a Cottage Hospital Nurse.

I must thank you for the weekly Hospital, which I appreciate very highly, but, in common with some other people, hope soon to see the novel turned out, so that the paper can be common property. I should not like my patients to think I read novels under cover of a useful periodical, although I am not strong-minded enough to do without light reading sometimes.

*** Why cannot The Hospital be common property, novel and all? There is no "under cover" about it, as nurses must have relaxation. We invite further correspondence on this subject of Novel or No Novel. What are the views and wishes of the majority of our readers?—[Ed. T. H.]

